# The efficacy of Salvia-ligustrazine and Ligustrazine in treating gestational hypertension: A systematic review and meta-analysis

**DOI:** 10.17305/bb.2024.10743

**Published:** 2024-12-01

**Authors:** Ruyi Ji, Qianrong Gan, Xinyao Shu, Ruitong Xu, Xinyu Huang, Tao Shen

**Affiliations:** 1 School of Basic Medical Sciences, Chengdu University of Traditional Chinese Medicine, Chengdu, China

**Keywords:** Pregnancy-induced hypertension syndrome (PIH), Salvia-ligustrazine, Ligustrazine, meta-analysis

## Abstract

Pregnancy-induced hypertension syndrome (PIH), a prevalent and critical condition, has garnered increasing attention due to its significant impact on maternal and fetal health outcomes. The conventional treatment approaches rely on magnesium sulfate and various antihypertensive drugs; however, the clinical efficacy of these treatments is limited, highlighting the need to explore alternative avenues for improvement. Recently, a growing number of clinical studies have investigated the use of Salvia-ligustrazine or Ligustrazine in combination with conventional therapy. A comprehensive synthesis and critical analysis of these studies is necessary to evaluate the efficacy and safety of Salvia-ligustrazine or Ligustrazine in treating PIH. We sought all articles published prior to December 2, 2023, from seven databases to identify randomized controlled trials (RCTs) that involved traditional Chinese medicine Salvia-ligustrazine or Ligustrazine in combination with Western medicines for the conventional treatment of PIH, according to predefined inclusion criteria. The studies were assessed using the Cochrane Risk of Bias tool (ROB2.0), and meta-analyses were conducted using Stata 15.0 statistical software. We analyzed 47 RCTs encompassing 4517 patients. The results demonstrated that combining Salvia-ligustrazine or Ligustrazine with Western medications was more efficacious than using Western medications alone. This combination improved the overall response rate, reduced the incidence of adverse pregnancy outcomes for mothers and infants, and decreased the occurrence of side effects associated with PIH treatment. While we evaluated the efficacy of traditional Chinese medicine injections of Salvia-ligustrazine or Ligustrazine alongside conventional Western treatments, our conclusions must be considered provisional due to potential bias and the limited availability of RCTs.

## Introduction

Pregnancy-induced hypertension (PIH) is one of the most prevalent obstetric conditions, characterized by high blood pressure, proteinuria, and other clinical signs that can endanger both maternal and infant health. In severe cases, such as eclampsia, PIH can lead to seizures, coma, and even death [1]. The conventional therapy primarily involves magnesium sulfate along with symptomatic treatments such as sedation and antihypertensive drugs. However, this approach has limited efficacy and is associated with side effects. Junxia et al. [2] found that prolonged, high-dose use of magnesium sulfate can inhibit maternal uterine contractions, prolong labor, and increase the risk of postpartum hemorrhage. Additionally, newborns may present with hypotonia and decreased responsiveness. Advancements in hypertension research have revealed that PIH involves organ ischemia due to vasospasm, leading to microcirculatory disturbances and increased blood viscosity [3]. According to traditional Chinese medicine (TCM) theory, the formation of blood stasis hinders the production of new blood [4]. Therefore, Chinese herbs that stimulate circulation and eliminate congested blood, such as Salvia miltiorrhiza and Rhizoma Ligustici Chuanxiong, are commonly used to treat this condition. Active ingredients extracted from these herbs, such as Salvia-ligustrazine and Ligustrazine (Chuanxiongzine), have been widely used in clinical settings as injections for the treatment of PIH in recent years. These injections promote circulation and reduce congestion [5]. Several clinical studies have demonstrated that the combined treatment with Chuanxiongzine injection significantly alleviates PIH compared to conventional treatments. Benefits include lowering blood pressure, reducing proteinuria, improving maternal and infant pregnancy outcomes, promoting coagulation, and reducing the incidence of drug-induced adverse events. Current meta-analyses on this treatment regimen have only focused on Chuanxiongzine [6], without considering the combination of Salvia-ligustrazine with conventional treatments. Additionally, these analyses often have limited sample sizes and few outcome indicators, making it difficult to confirm the efficacy of the regimen. Therefore, to provide more robust evidence on the role of Salvia-ligustrazine or Ligustrazine in combination with conventional Western drug regimens for PIH, we conducted a comprehensive meta-analysis of relevant articles published in recent years to confirm the efficacy and safety of this treatment approach.

## Materials and methods

This report was designed and conducted in accordance with the Preferred Reporting Items for Systematic Reviews and Meta-Analyses (PRISMA) guidelines and is registered on the PROSPERO website for meta-analysis (CRD42024496232).

### Literature search strategy

We searched the following databases: PubMed, Embase, Cochrane Library, Web of Science, China National Knowledge Infrastructure (CNKI), Wanfang Database, and VIP Database. The literature search was limited to studies published up to December 2, 2023, in English and Chinese languages. The search involved a combination of subject terms and free terms, including the following medical subject headings: Hypertension, Pregnancy-Induced, Salvia-ligustrazine, and Ligustrazine. Detailed search strategies are provided in Supplementary Text 1.

### Study inclusion and exclusion criteria

#### Inclusion criteria (Table 1)

P (participants): Individuals with hypertensive disorders associated with pregnancy, including gestational hypertension, pre-eclampsia/eclampsia, chronic hypertension, and chronic hypertension with superimposed pre-eclampsia. Subjects were diagnosed based on the Guidelines for the Diagnosis and Treatment of Hypertensive Diseases in Pregnancy (2020 edition) [7].

I (intervention): The treatment group received routine treatment with Salvia-ligustrazine or Ligustrazine combined with Western medicines.

C (control): The control group received only conventional Western medicines, including but not limited to magnesium sulfate, nifedipine, labetalol, and other sedative antihypertensive treatments.

O (outcomes): Primary outcome measures: changes in blood pressure (systolic blood pressure, SBP; diastolic blood pressure, DBP), maternal and infant pregnancy outcomes, overall response rate (ORR). Secondary outcome measures: Incidence of adverse effects, urine protein levels, coagulation indicators (Activated partial thromboplastin time, APTT; Prothrombin time, PT).

S (study design): Randomized controlled trials (RCTs).

#### Exclusion criteria

The exclusion of subjects was as below: 1) Duplicate studies; 2) Noncompliance with the intervention; and 3) Lack of valid data. Specifically, literature lacking outcome indicators distinct from those listed enumerated under “O (outcome)” will not be included.

Two assessors (RYJ and GQR) independently reviewed the literature based on the above criteria, with any inconsistencies resolved through discussion or by consulting a third reviewer (ZYZ).

### Extraction of data and assessment of study quality

Two reviewers (R.Y.J. and G.Q.R.) independently extracted data from the final set of included articles, which included information on the first author, year of publication, country, interventions and controls, treatment duration, basic information about the study population, and outcome measures.

The Cochrane Risk of Bias Assessment Tool (ROB 2.0) was used to evaluate the risk of bias in the selected articles. ROB 2.0 assesses five aspects: bias arising from the random allocation process, bias due to deviations from established procedures, bias arising from missing outcome data, bias related to outcome measures, and bias resulting from selective reporting of results. Each study was independently assessed by two reviewers using ROB 2.0, with any disagreements resolved by a third reviewer (ZYZ). The results are presented as risk of bias plots.

### Statistical analysis

The primary outcome indicators were changes in blood pressure, including SBP and DBP, maternal and infant pregnancy outcomes, and ORR, while the secondary outcome indicators were urine protein levels, coagulation indices (APTT; PT), and the occurrence of adverse reactions.

Meta-analysis was performed using Stata15.0. Weighted mean differences (WMD) were calculated for continuous data, with 95% confidence intervals (CI) reported when the same scale was used. For dichotomous variables, meta-analysis was performed using relative risk (RR) to indicate effect size. Heterogeneity between studies was assessed using the *Q* test for X^2^ and I^2^ statistic. When heterogeneity across studies was not significant (I^2^ <50% and *P* > 0.1), the Mantel–Haenszel model was used for meta-analysis. Conversely, a random-effects model was employed.

To explore the extent and sources of heterogeneity among studies, subgroup analyses and regression analyses of Salvia-ligustrazine or Ligustrazine combined with conventional treatment were conducted based on treatment duration. Sensitivity analysis was performed to assess the robustness of the meta-analysis results, and publication bias in the included literature was evaluated using funnel plots, with statistical testing conducted using the Egger or Begg method (for studies with *n* ≥ 8). For results showing with significant publication bias, a cut-and-patch approach was used to assess its impact on the findings.

## Results

### Literature search

**Figure 1.**
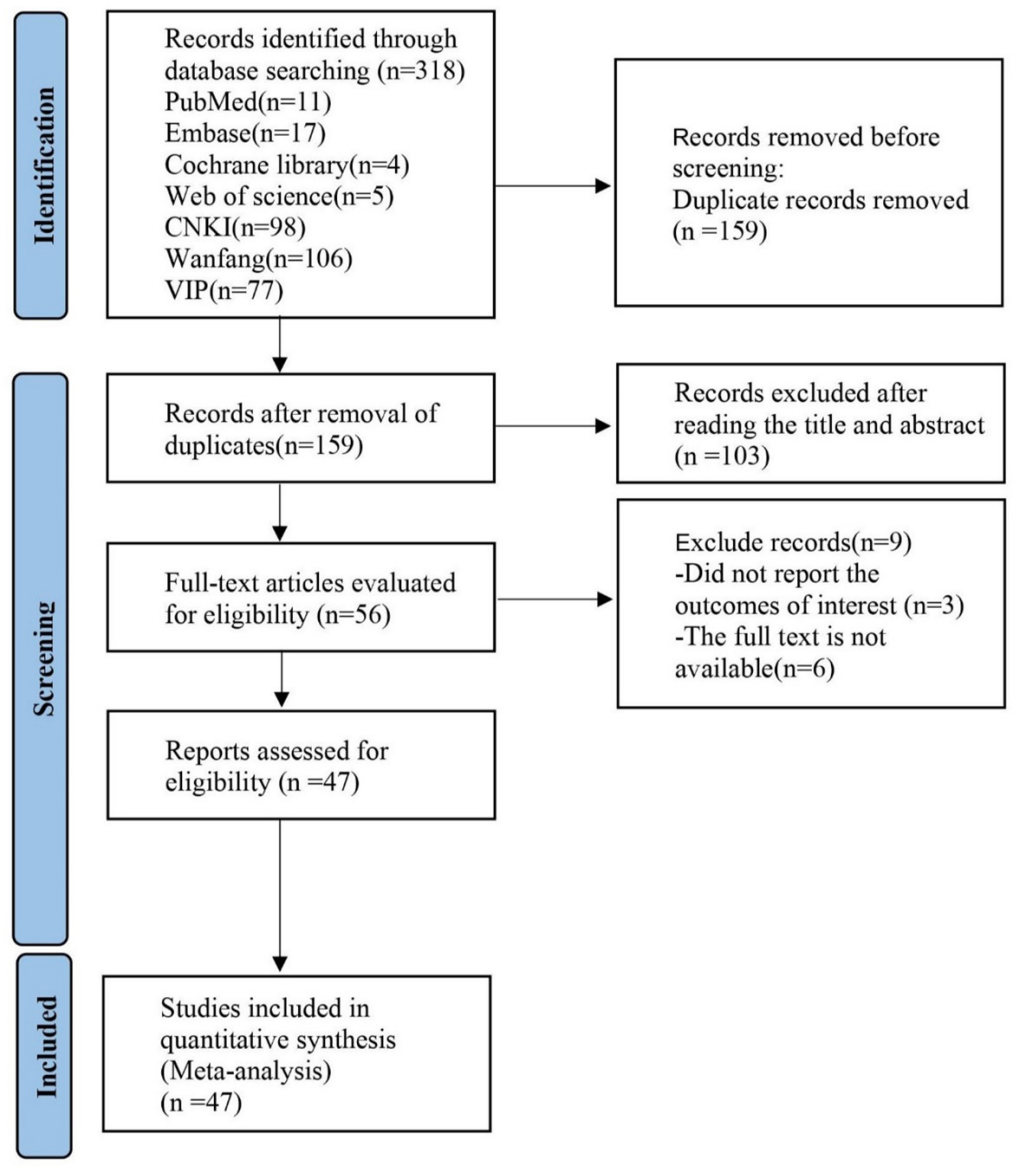
**Flowchart of searching and screening for the studies.** CNKI: China National Knowledge Infrastructure.

The initial database search yielded 318 articles. After removing duplicates and excluding articles that did not meet the inclusion criteria, 63 articles were thoroughly reviewed. Ultimately, 47 studies [5, 8–53] were identified as eligible for meta-analysis. The literature screening process is depicted in Figure 1.

**Table 1:** PICOS

P (participants)		Individuals with hypertensive disorders associated with pregnancy
I (intervention)		Salvia-ligustrazine or Ligustrazine combined with Western medicines
C (control)		Only conventional western medicines
O (outcomes)	Primary	SBP and DBP; pregnancy outcomes; ORR
	Secondary	Incidence of adverse effects; urine proteins; APTT, PT
S (study design)		RCTs

SBP: Systolic blood pressure; DBP: Diastolic blood pressure; ORR: Overall response rate; APTT: Activated partial thromboplastin time; PT: Prothrombin time; RCTs: Randomized controlled trials.

### The characteristics of included studies

The 47 included studies were conducted in China and involved a total of 4517 female patients, with an average age ranging from 21 to 42 years. The fundamental attributes of the included studies are presented in Table 1.

### Risk of bias of the included studies

**Figure 2.**
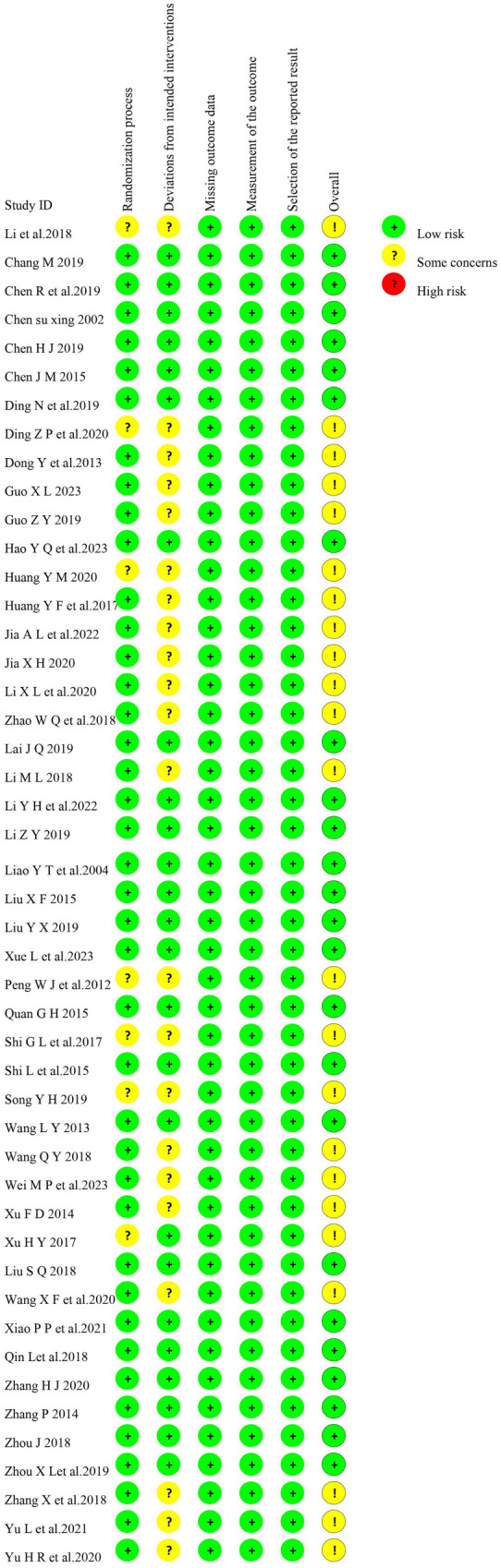
The risk of bias assessment.

Figure 2 shows the results of the risk of bias assessment for the 47 included trials. There was a potential risk in 23 studies [5, 10, 11, 13, 14, 20–22, 25, 27–29, 36, 38, 41–46, 48, 50, 51] due to missing random assignment, missing subgroup concealment, or lack of blinding. The remaining 24 studies [8, 9, 12, 15–19, 23, 24, 26, 30–35, 37, 39, 40, 47, 49, 52, 53] were deemed to have a low risk of bias. All studies showed minimal risk for missing outcome data, missing outcome measures, and selective reporting. Overall, the included literature presented a low risk of bias.

### Meta-analysis results

#### Main outcome indicators

*Changes in blood pressure: SBP, DBP*

**Figure 3.**
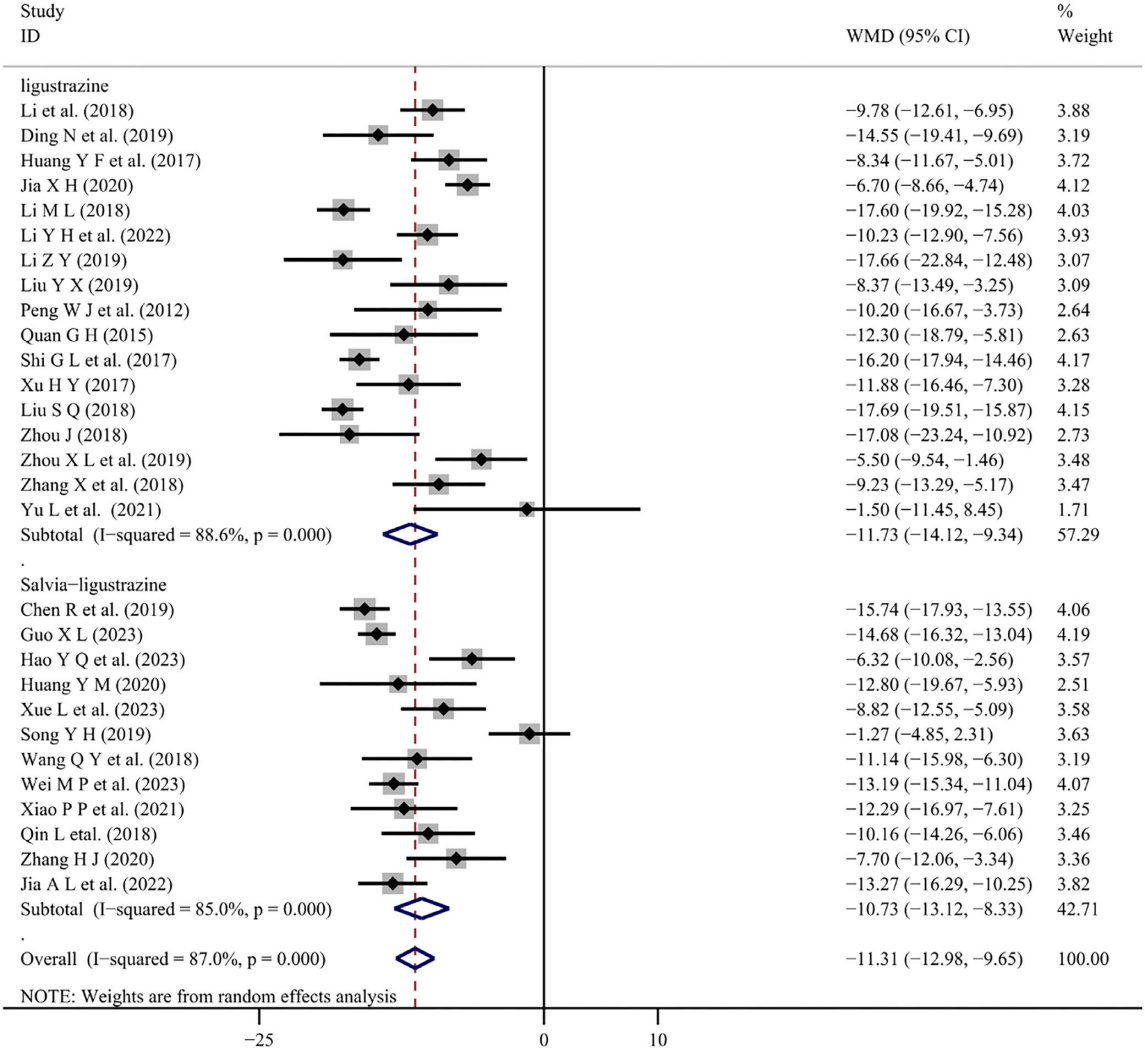
**Forest plot of SBP.** SBP changes in PIH patients treated with Salvia-ligustrazine or Ligustrazine injection combined with conventional treatment. SBP: Systolic blood pressure; PIH: Pregnancy-induced hypertension; WMD: Weighted mean differences; CI: Confidence intervals.

**Figure 4.**
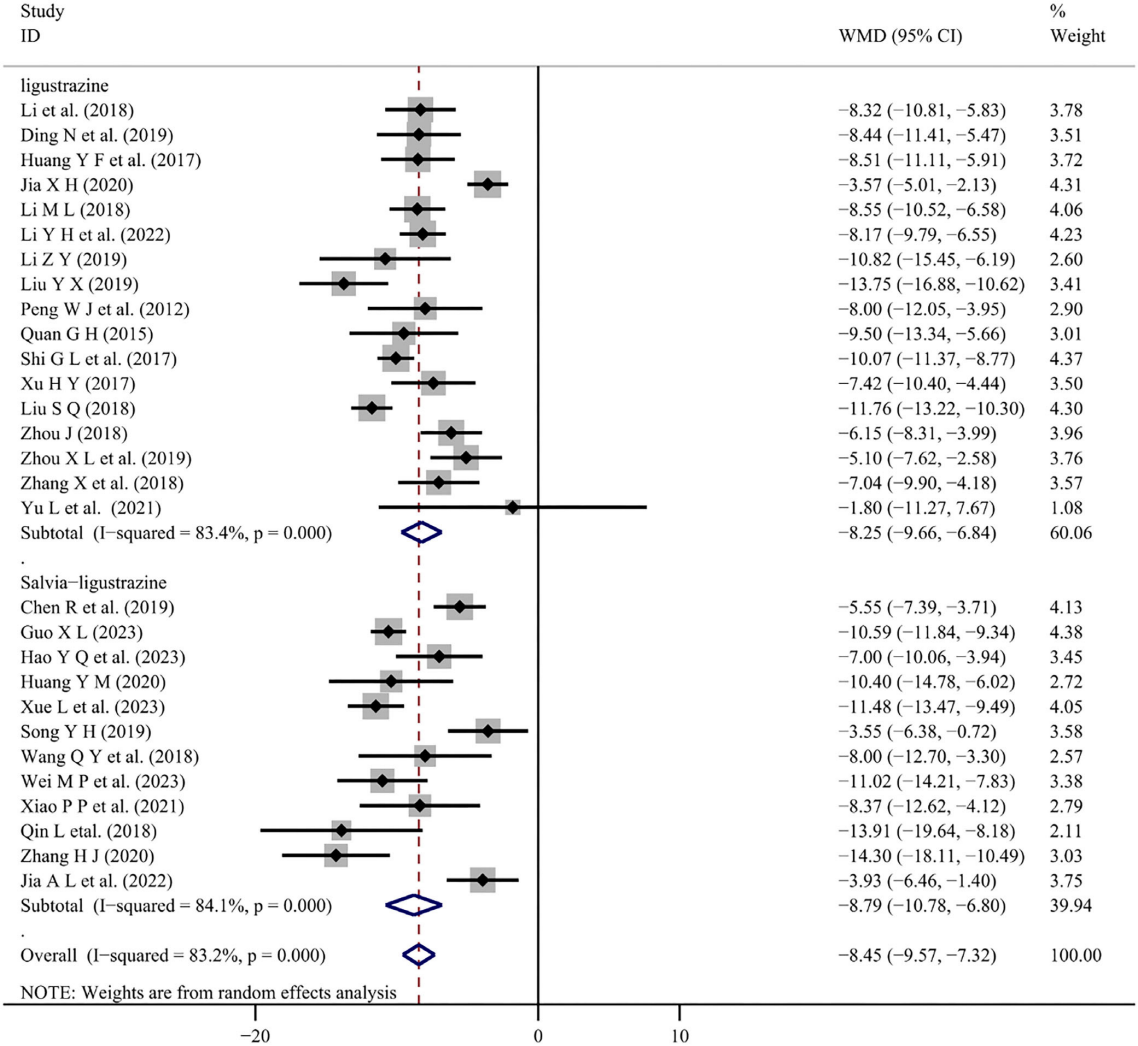
**Forest plot of DBP.** The diastolic blood pressure changes. WMD: Weighted mean differences; CI: Confidence intervals.

There were 12 studies [5, 24, 34, 36, 40, 44, 47, 48, 50–53] and 17 studies [11, 16, 20–23, 25, 26, 28, 29, 33, 35, 37, 39, 42, 46, 49] reported changes in blood pressure with conventional treatment combined with Salvia-ligustrazine or Chuanxiongzine, respectively. Both SBP and DBP changes were analyzed using random-effects meta-analysis (*I*^2^ ═ 85.0%, *P* < 0.001); (*I*^2^ ═ 83.2.0%, *P* < 0.001). The results indicate that combining Salvia-ligustrazine injection significantly reduced SBP (WMD ═ −10.73; 95% CI: −13.12 to −8.33; *P* < 0.001) and DBP (WMD ═ −8.79; 95% CI: −10.78 to −6.80; *P* < 0.001) compared to conventional treatment. Treatment with Chuanxiongzine injection also significantly reduced SBP (WMD ═ −11.73; 95% CI: −14.12 to −9.34; *P* < 0.001) and DBP (WMD ═ −8.25; 95% CI: −9.66 to −6.84; *P* < 0.001) (Figures 3 and 4).

*Pregnancy outcome-mother*

**Figure 5.**
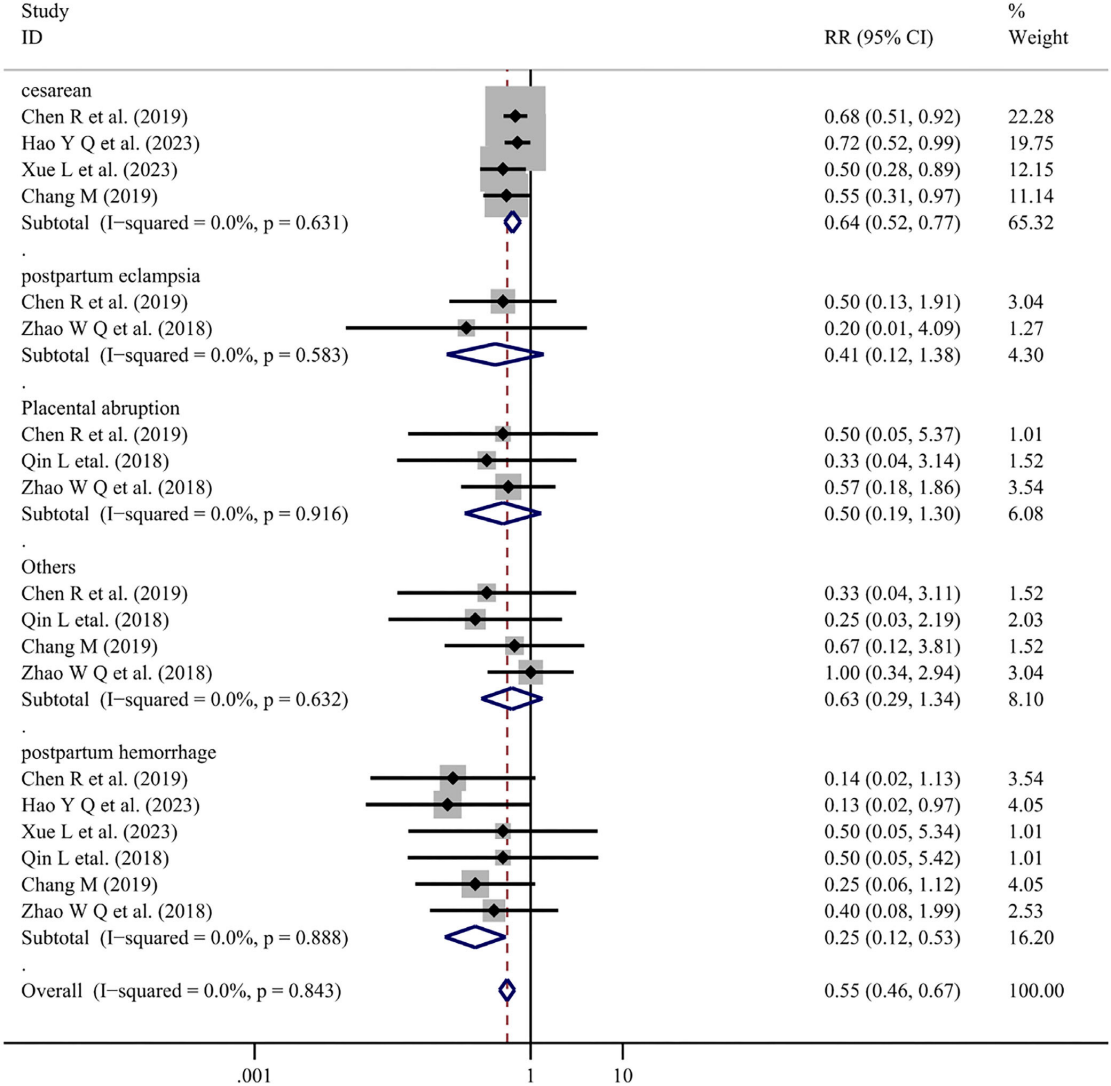
**Forest plot of Salvia-ligustrazine on pregnancy outcomes for mothers.** RR: Relative risk; CI: Confidence intervals.

Six studies [24, 27, 32, 34, 52, 53] reported pregnancy outcomes with Salvia-ligustrazine combined with conventional therapy. The heterogeneity test for Salvia-ligustrazine treatment showed no significant heterogeneity among the included studies (*I*^2^ ═ 0.0%, *P* ═ 0.843), leading to the use of a fixed-effects model. The results showed that the Salvia-ligustrazine combination reduced the risk of several pregnancy outcomes compared with conventional treatment. [Cesarean (RR ═ 0.64; 95% CI: 0.52–0.77; *P* < 0.001), Postpartum eclampsia (RR ═ 0.41; 95% CI: 0.12–1.38; *P* ═ 0.151), Placental abruption (RR ═ 0.50; 95% CI: 0.19–1.30; *P* ═ 0.154), Postpartum hemorrhage (RR ═ 0.25; 95% CI: 0.12–0.53; *P* < 0.001), Others (RR ═ 0.63; 95% CI: 0.29–1.34; *P* ═ 0.229)] However, there was no significant difference in the incidence of postpartum eclampsia, placental abruption, or other outcomes when comparing TCM combination therapy with Western medicine alone. The small sample size in the studies involving in Salvia-ligustrazine Combination Therapy, may account for this (Figure 5).

**Figure 6.**
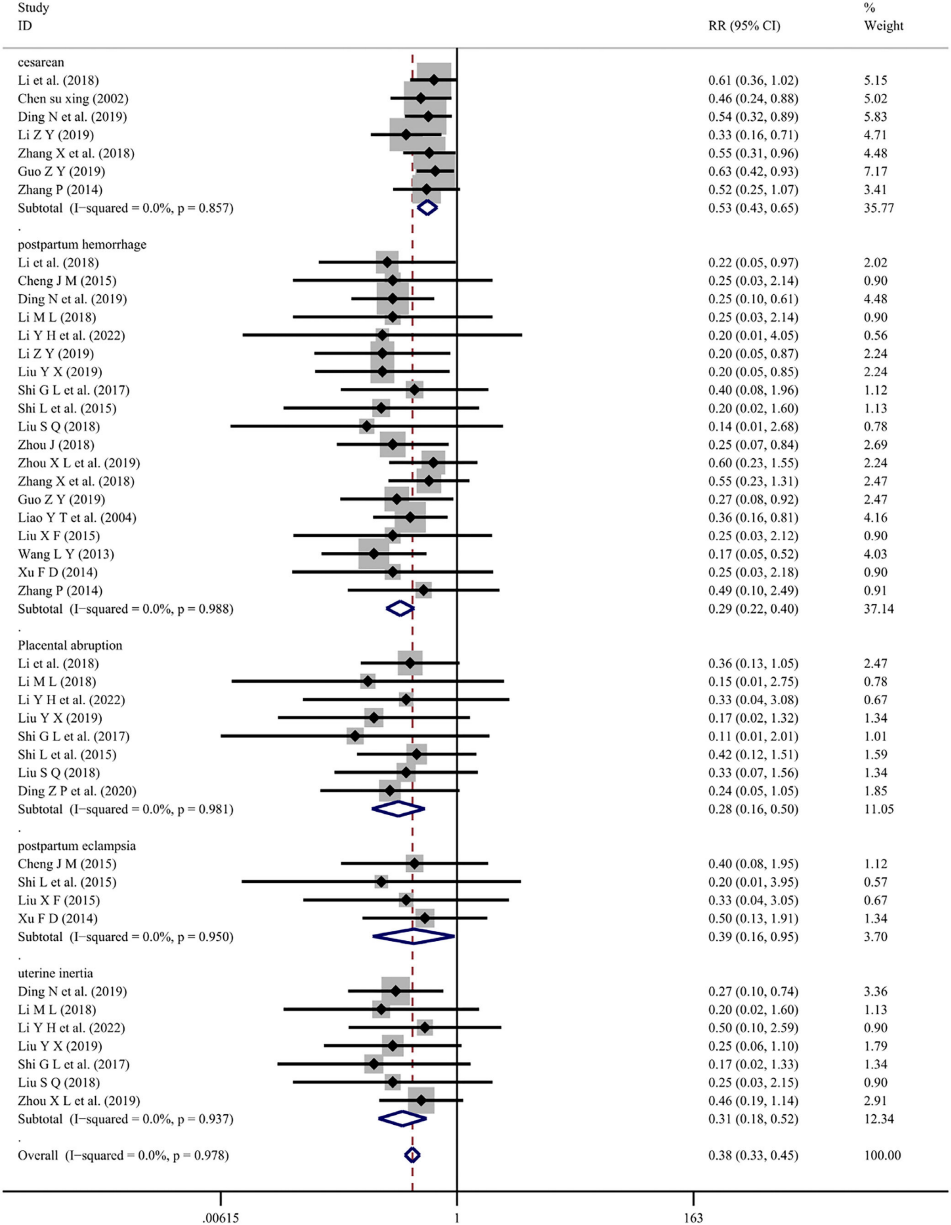
**Forest plot of Ligustrazine on pregnancy outcomes for mothers.** RR: Relative risk; CI: Confidence intervals.

Twenty-one studies [8, 9, 12, 14, 15, 17–20, 23, 25, 26, 28, 29, 33, 35, 37–39, 45, 49] reported pregnancy outcomes with Chuanxiongzine combined with conventional therapy. The heterogeneity test was nonsignificant (*I*^2^ ═ 0.0%, *P* ═ 0.978), so a fixed-effects model was employed to summarize the results. This analysis indicated that the combination of Chuanxiongzine reduced the risk of several maternal pregnancy outcomes compared with conventional Western drug therapy [Cesarean (RR ═ 0.53; 95% CI: 0.43–0.65; *P* < 0.001), Postpartum hemorrhage (RR ═ 0.29; 95% CI: 0.22–0.40; *P* < 0.001), Placental abruption (RR ═ 0.28; 95% CI: 0.16–0.50; *P* < 0.001), Postpartum eclampsia (RR ═ 0.39; 95% CI: 0.16–0.95; *P* ═ 0.038), and Uterine inertia (RR ═ 0.31; 95% CI: 0.18–0.52; *P* < 0.001) (Figure 6).

*Pregnancy outcome-fetus*

**Figure 7.**
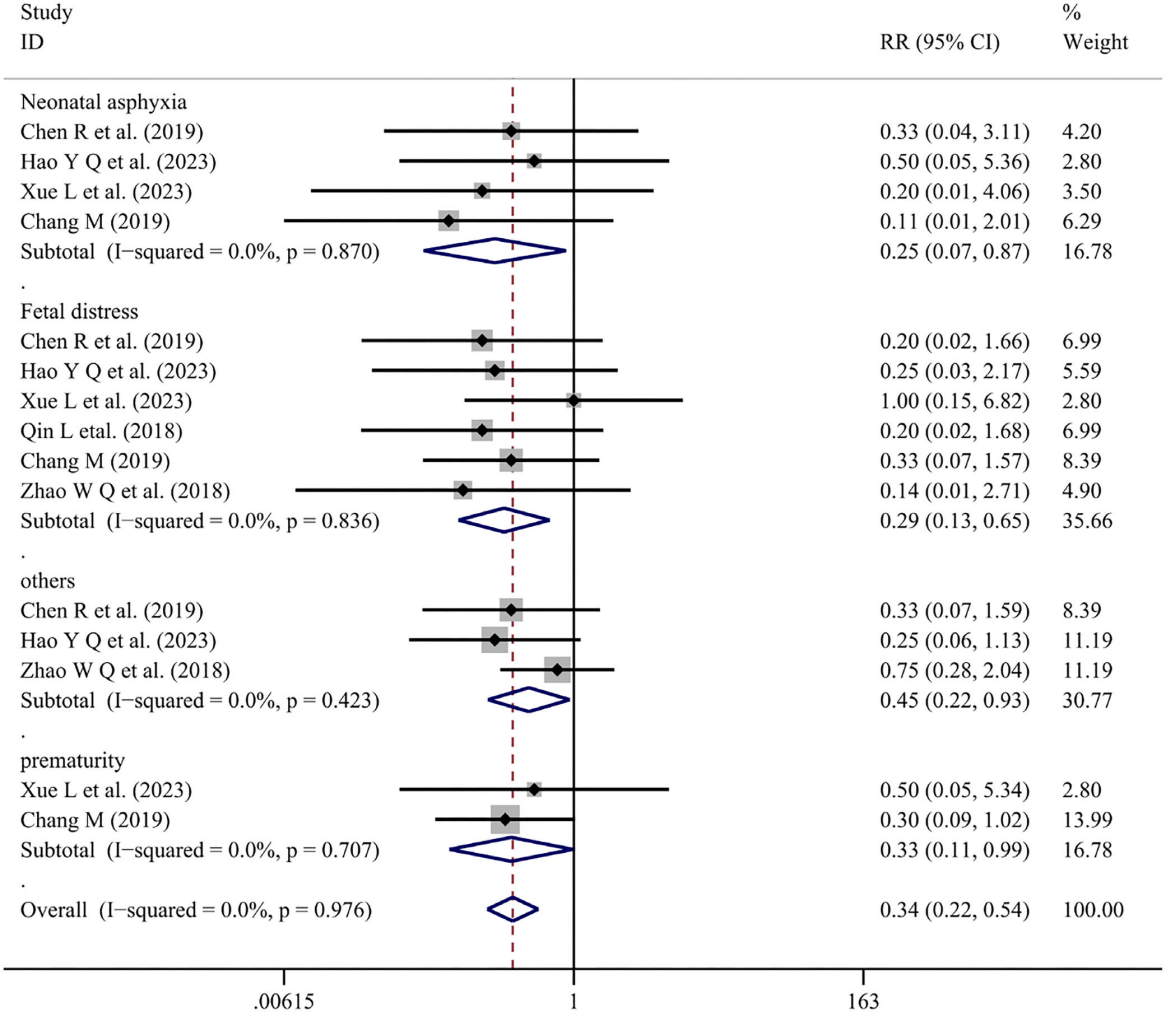
**Forest plot of Salvia-ligustrazine on pregnancy outcomes for the fetus.** RR: Relative risk; CI: Confidence intervals.

Six articles [24, 27, 32, 34, 52, 53] reported fetal pregnancy outcomes with Salvia-ligustrazine combined with conventional therapy. The heterogeneity test was nonsignificant (*I*^2^=0.0%, *P* ═ 0.976), leading to the use of a fixed-effects model. The findings indicated that combined Salvia-ligustrazine treatment reduced the risk of several fetal pregnancy outcomes compared with Western medicine alone [Neonatal asphyxia (RR ═ 0.25; 95% CI: 0.07–0.87; *P* ═ 0.030), Fetal distress (RR ═ 0.29; 95% CI: 0.13–0.65; *P* ═ 0.003), Others (RR ═ 0.45; 95% CI: 0.22–0.93; *P* ═ 0.031), and Prematurity (RR ═ 0.33; 95% CI: 0.11–0.99; *P* ═ 0.047)] (Figure 7).

**Figure 8.**
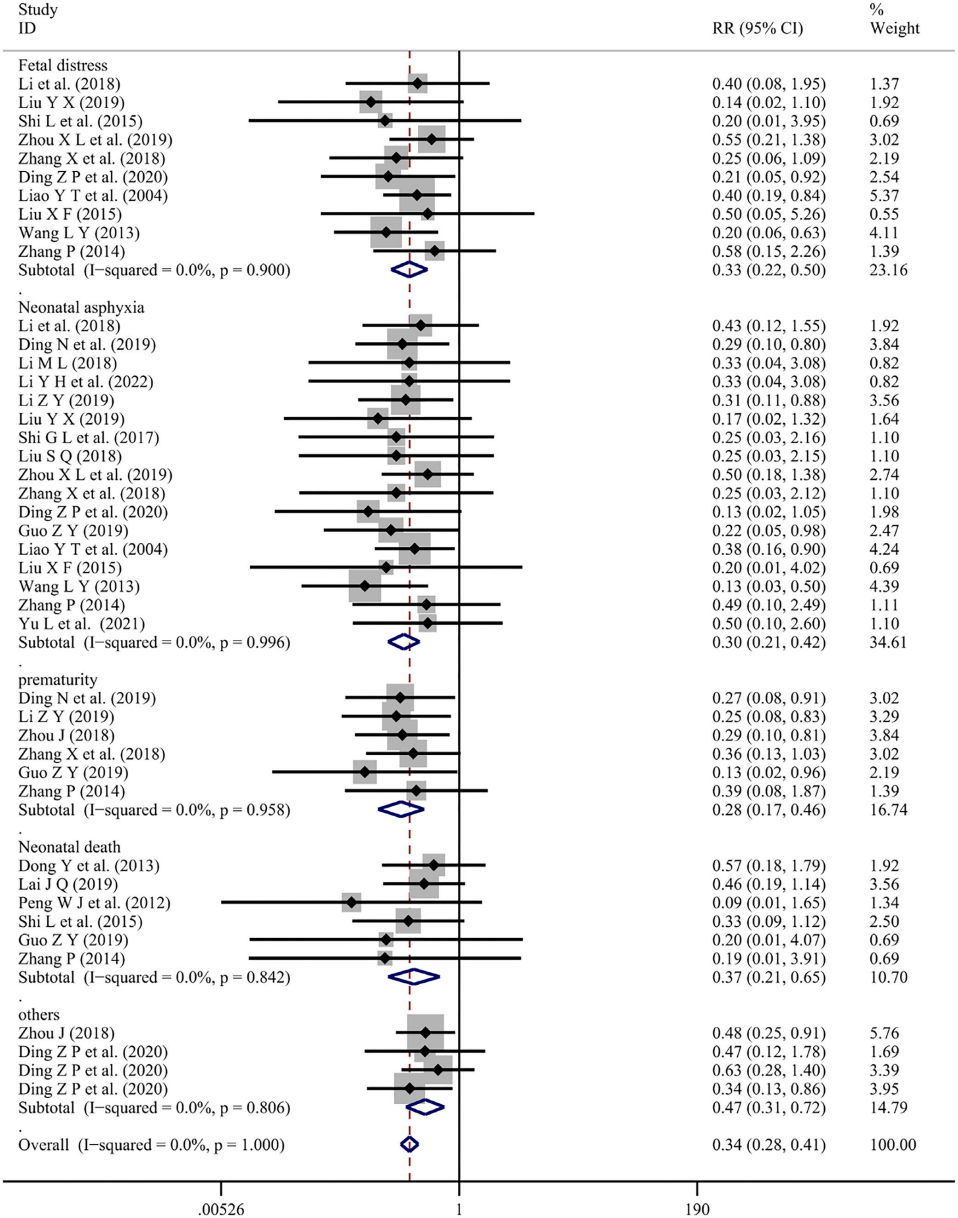
**Forest plot of Ligustrazine on pregnancy outcomes for the fetus.** RR: Relative risk; CI: Confidence intervals.

Twenty-two articles [9, 11–13, 15, 18–20, 23, 25, 26, 28, 29, 31, 33, 35, 37–39, 45, 46, 49] reported fetal pregnancy outcomes with Chuanxiongzine combined conventional therapy (*I*^2^ ═ 0.0%, *P* ═ 1.000). The results were integrated via a fixed-effects model, revealing that combined Ligustrazine treatment reduced the risk of several fetal pregnancy outcomes compared to conventional Western medicine [Fetal distress (RR ═ 0.33; 95% CI: 0.22–0.50; *P* < 0.001), Neonatal asphyxia (RR ═ 0.30; 95% CI: 0.21–0.42; *P* < 0.001), Prematurity (RR ═ 0.28; 95% CI: 0.17–0.46; *P* < 0.001), Neonatal death (RR ═ 0.37; 95% CI: 0.21–0.65; *P* ═ 0.001), and Others (RR ═ 0.47; 95% CI: 0.31–0.72; *P* < 0.001)] (Figure 8).

*Overall response rate*

**Figure 9.**
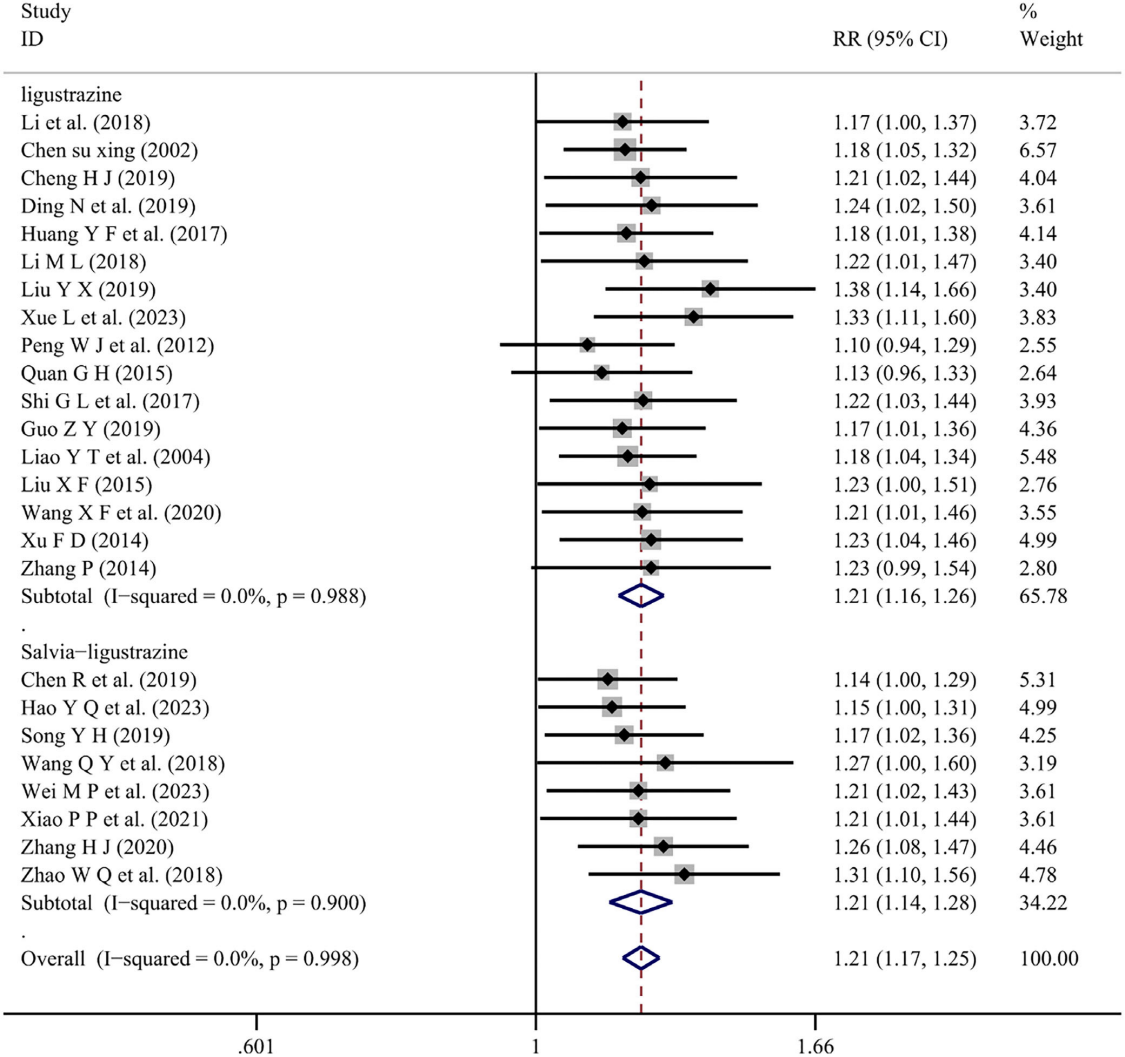
**Forest plot of overall response rate.** RR: Relative risk; CI: Confidence intervals.

Eight studies [5, 27, 34, 36, 40, 47, 50, 53] and 17 studies [8, 9, 11, 14–16, 19, 20, 22, 25, 28, 30, 33, 37, 38, 43, 52] reported the ORR of conventional Western medicine combined with Salvia-ligustrazine or Ligustrazine, respectively. The heterogeneity test analysis showed no significance (*I*^2^=0.0%, *P* ═ 0.998). Results indicated that Salvia-ligustrazine or Ligustrazine combination treatment significantly increased the overall response rate compared to conventional Western medicine treatment [Salvia, Ligustrazine (RR ═ 1.21; 95% CI: 0.17–1.25; *P* < 0.001), Ligustrazine (RR ═ 1.21; 95% CI: 1.16–1.26; *P* < 0.001)] (Figure 9).

#### Secondary outcome indicators

*Urine protein*

**Figure 10.**
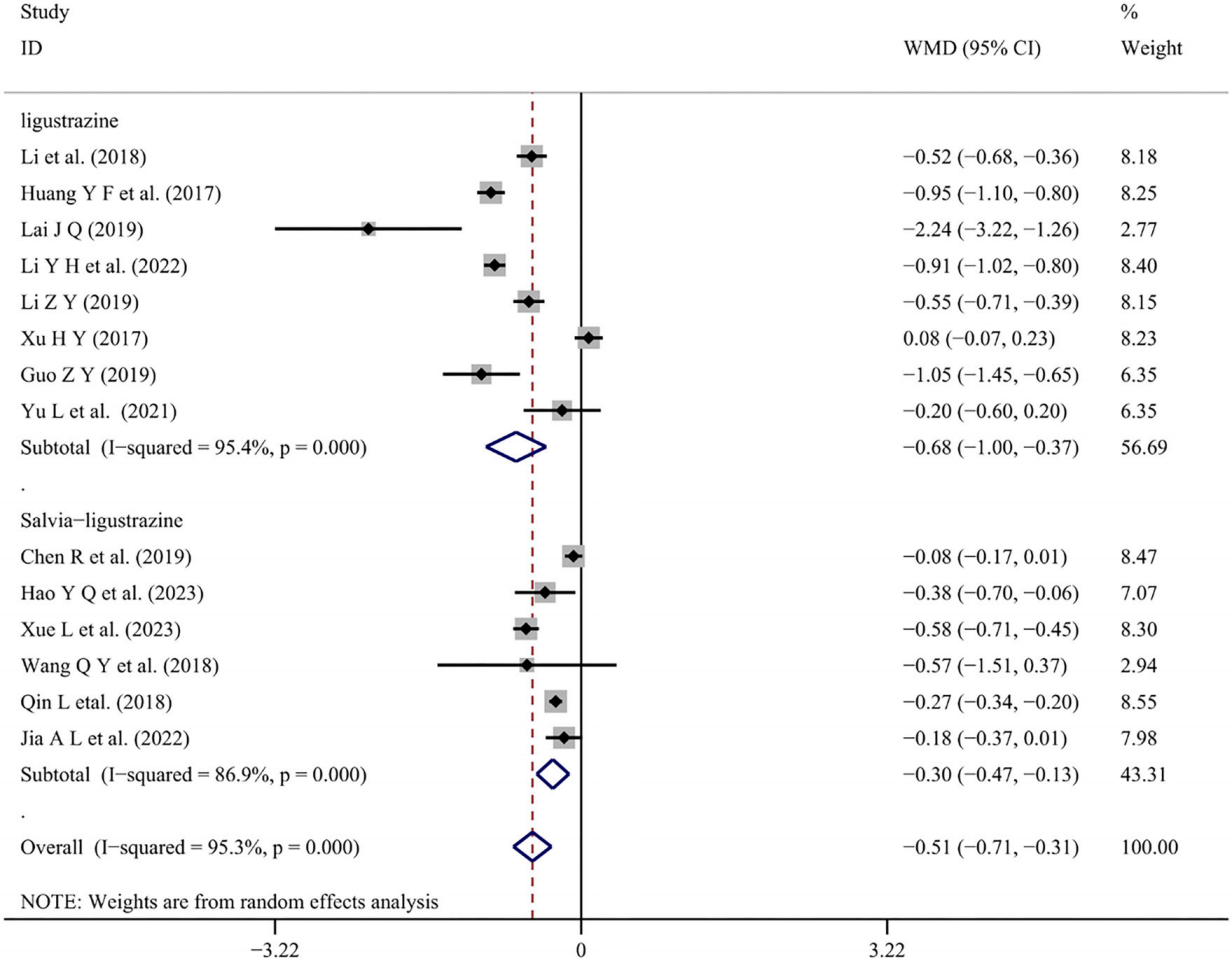
**Forest plot of urine protein.** WMD: Weighted mean differences; CI: Confidence intervals.

Six studies [5, 24, 34, 48, 52, 53] and eight studies [21, 22, 28, 31, 38, 39, 46, 49] reported changes in 24-h urine protein levels with conventional treatment combined with Salvia-ligustrazine or Ligustrazine, respectively. A random-effects model (I^2^ ═ 95.3%, *P* < 0.001) was applied. The meta-analysis revealed that both combined treatments with Salvia-ligustrazine and Ligustrazine significantly reduced patients’ 24-h urine protein levels (WMD ═ −0.30; 95% CI: −0.47 to −0.13; *P* < 0.001), (WMD ═ −0.68; 95% CI: −1.00 to −0.37; *P* < 0.001) (Figure 10).

**Figure 11.**
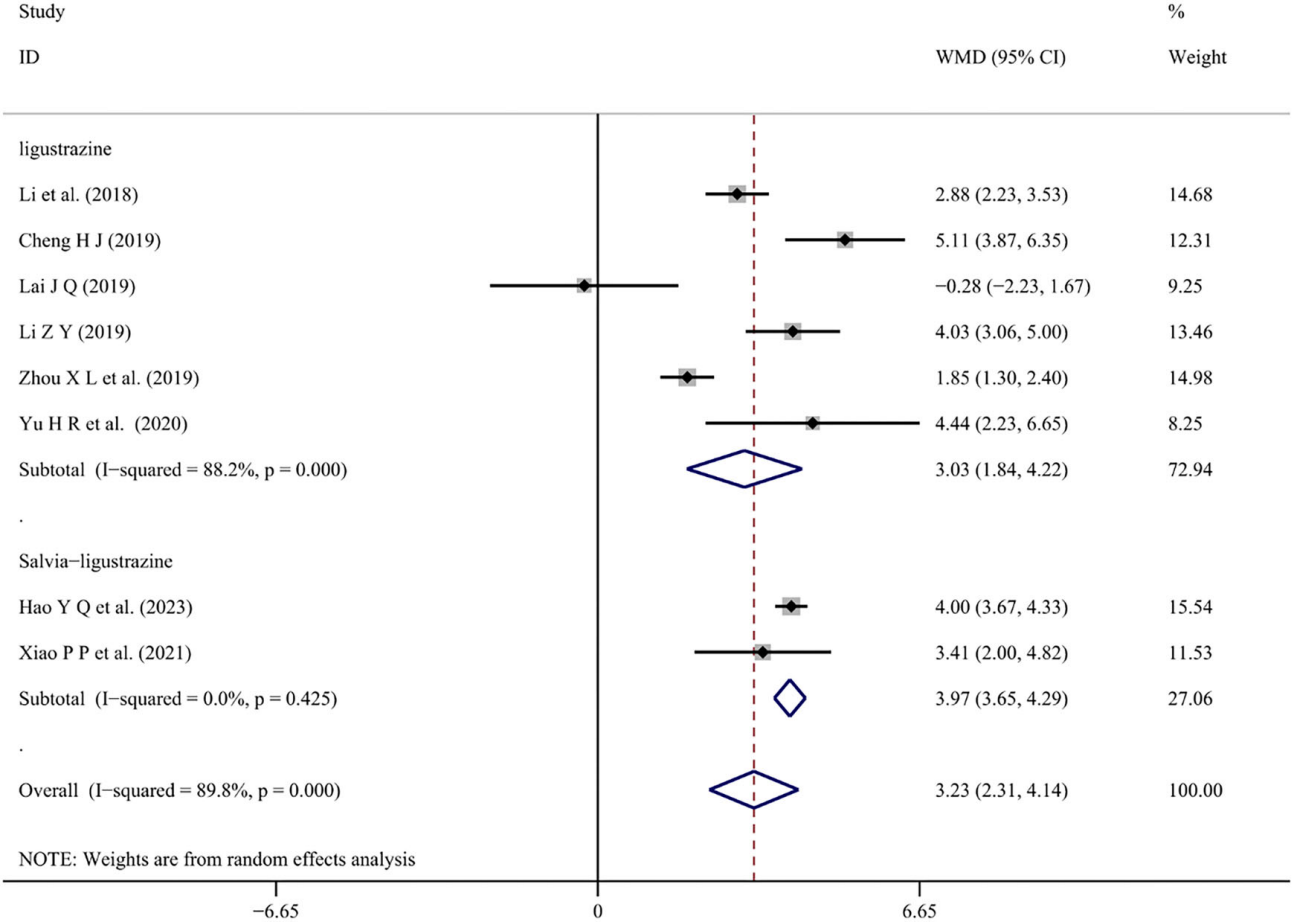
**Forest plot of activated partial thromboplastin time.** WMD: Weighted mean differences; CI: Confidence intervals.

*Coagulation function (APTT, PT)*

**Figure 12.**
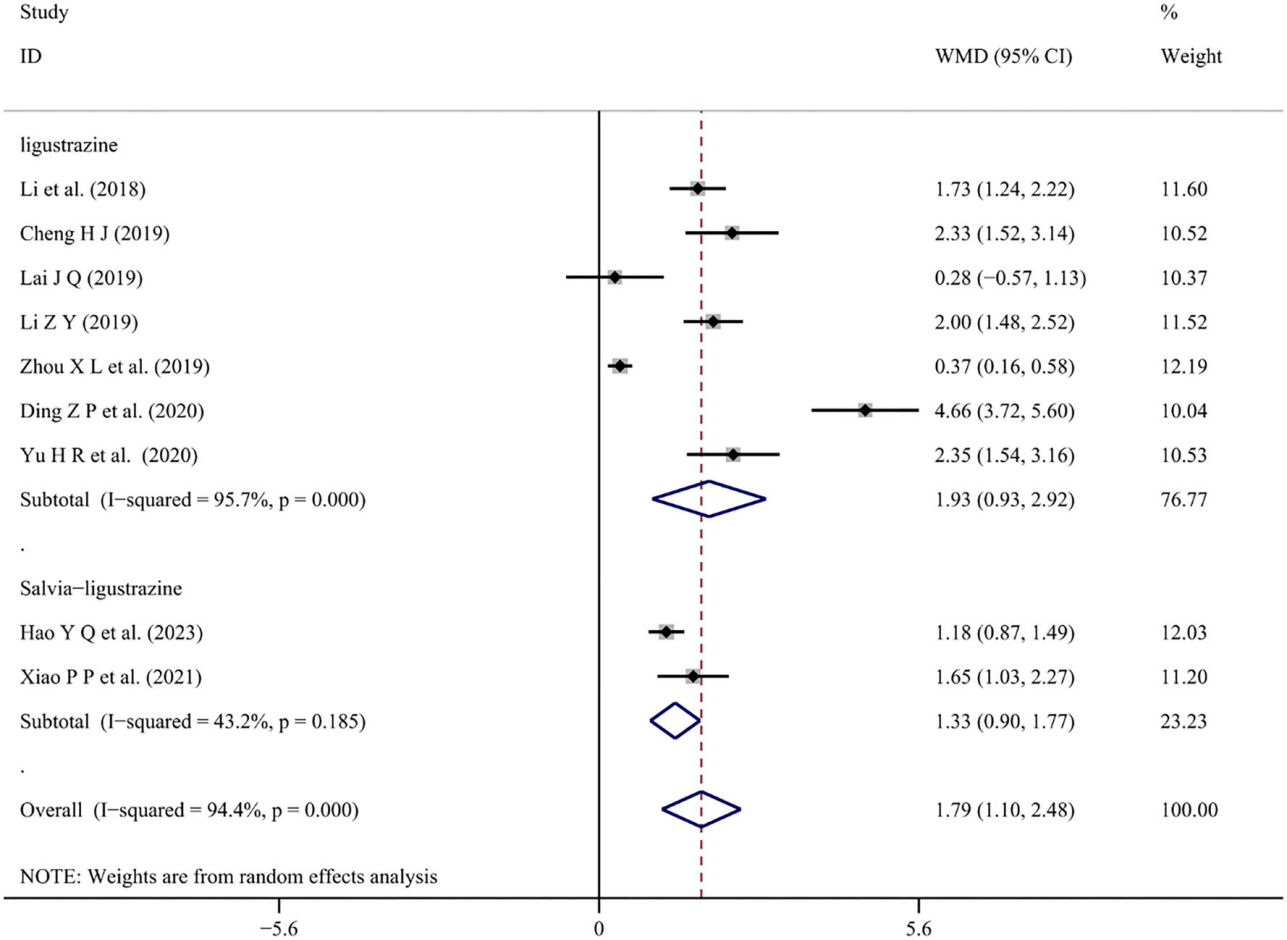
**Forest plot of prothrombin time.** WMD: Weighted mean differences; CI: Confidence intervals.

Changes in APTT and PT values with conventional treatment combined with Chinese medicine injections were reported in eight [28, 30, 31, 35, 39, 41, 47, 53] and nine studies [28, 30, 31, 35, 39, 41, 45, 47, 53], respectively. These changes were meta-analyzed using a random-effects model (*I*^2^ ═ 89.8%, *P* < 0.001), (*I*^2^ ═ 94.4%, *P* < 0.001). The meta-analysis showed that combined treatment with Salvia-ligustrazine or Ligustrazine increased both APTT and PT values in patients’ blood (WMD ═ 3.23; 95% CI: 2.31–4.14; *P* < 0.001), (WMD ═ 1.79; 95% CI: −1.10 to 2.48; *P* < 0.001) (Figures 11 and 12).

**Figure 13.**
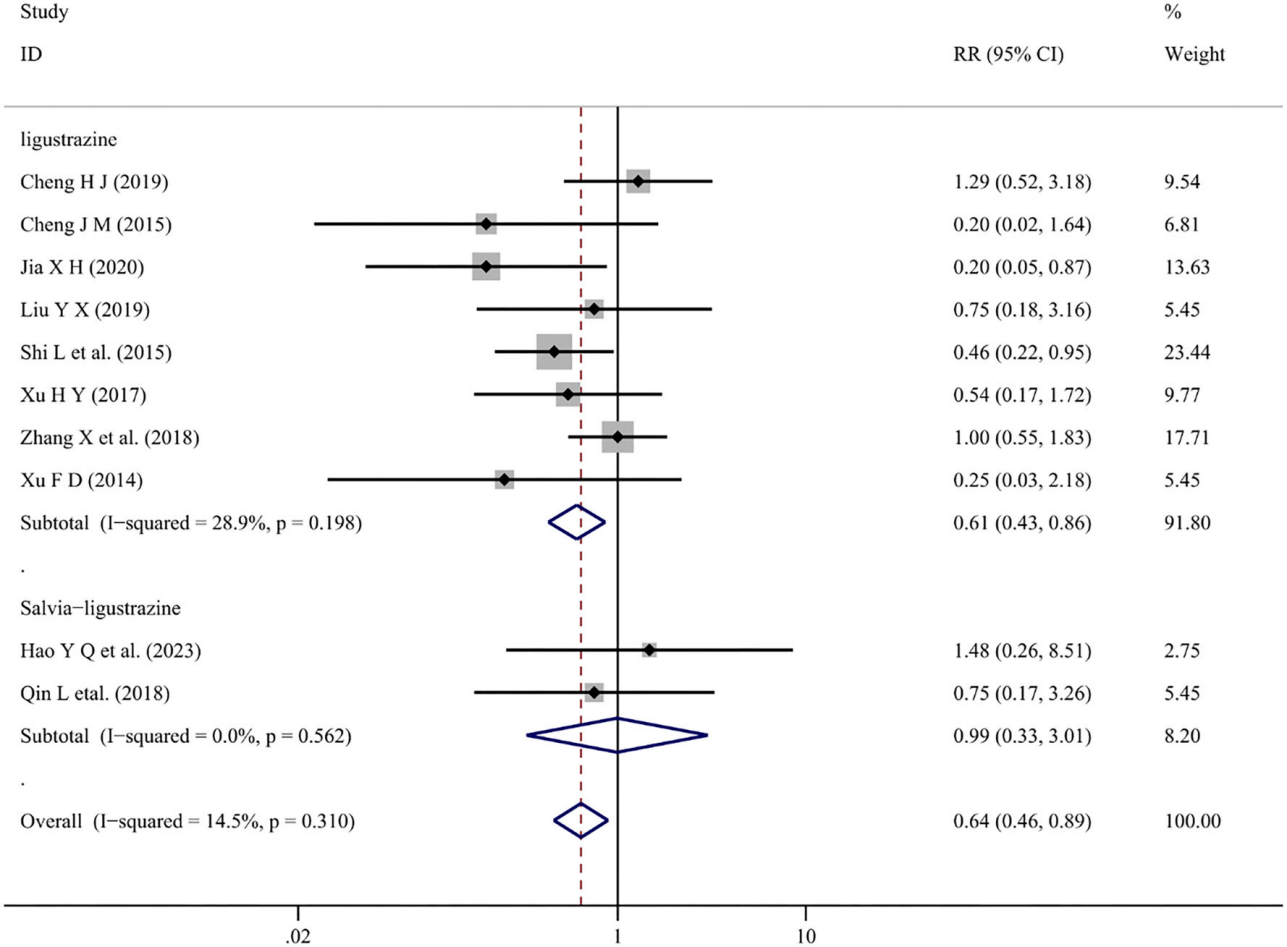
**Forest plot of untoward reaction.** RR: Relative risk; CI: Confidence intervals.

*Untoward reaction*

Ten articles [14, 17, 18, 21, 24, 29, 30, 37, 42, 53] reported adverse events associated with conventional treatment combined with Salvia-ligustrazine or Ligustrazine. The adverse events included headache, dizziness, chest tightness, and impaired liver and kidney function (*I*^2^ ═ 14.5%, *P* ═ 0.310). The findings indicated that, compared with conventional treatment alone, combined Chinese medicine injections reduced the number of adverse reactions in patients (WMD ═ 0.64; 95% CI: 0.46–0.89; *P* ═ 0.008) (Figure 13).

*Subgroup analysis*

**Figure 14.**
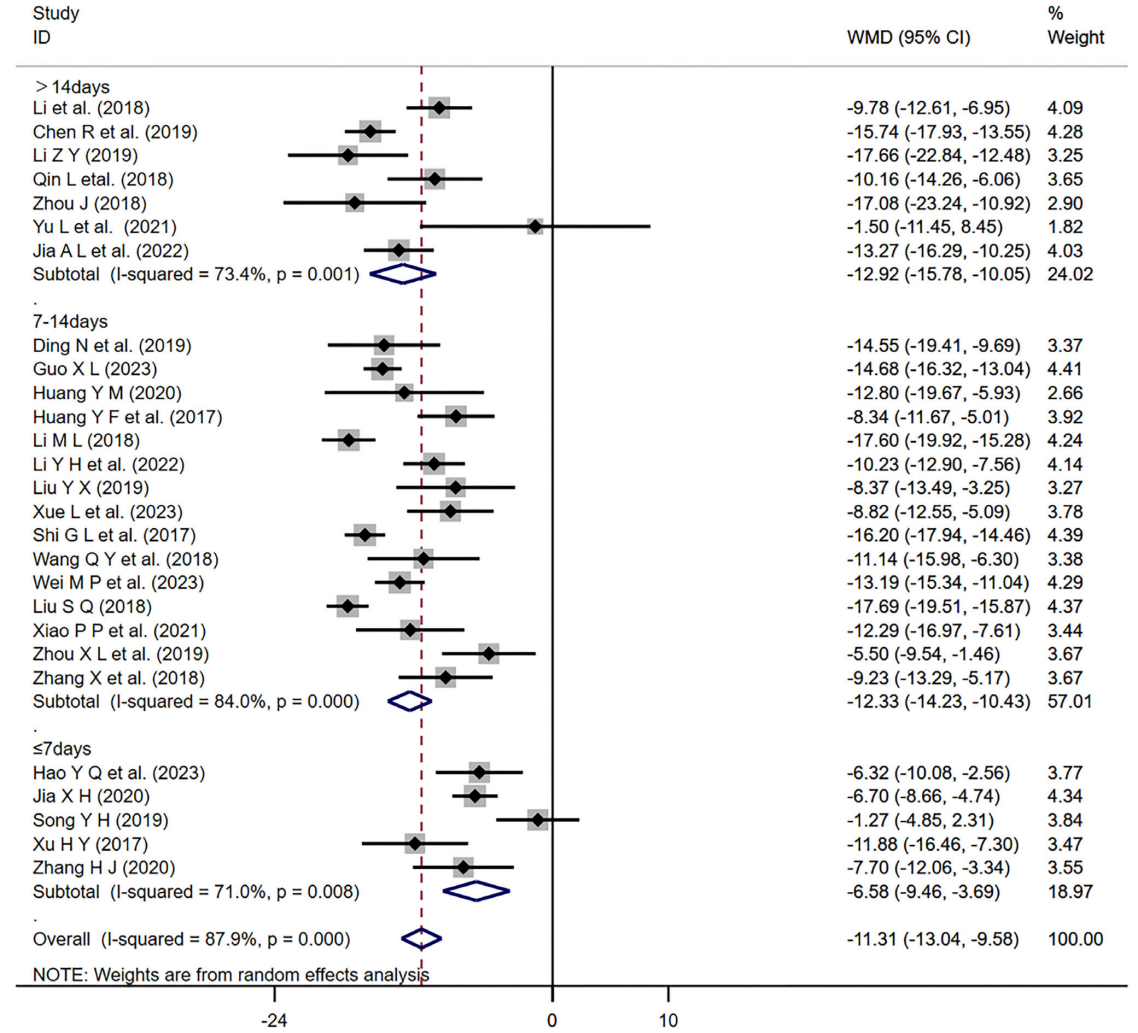
**Forest plot of treatment duration subgroup analysis of SBP.** SBP: Systolic blood pressure; WMD: Weighted mean differences; CI: Confidence intervals.

**Figure 15.**
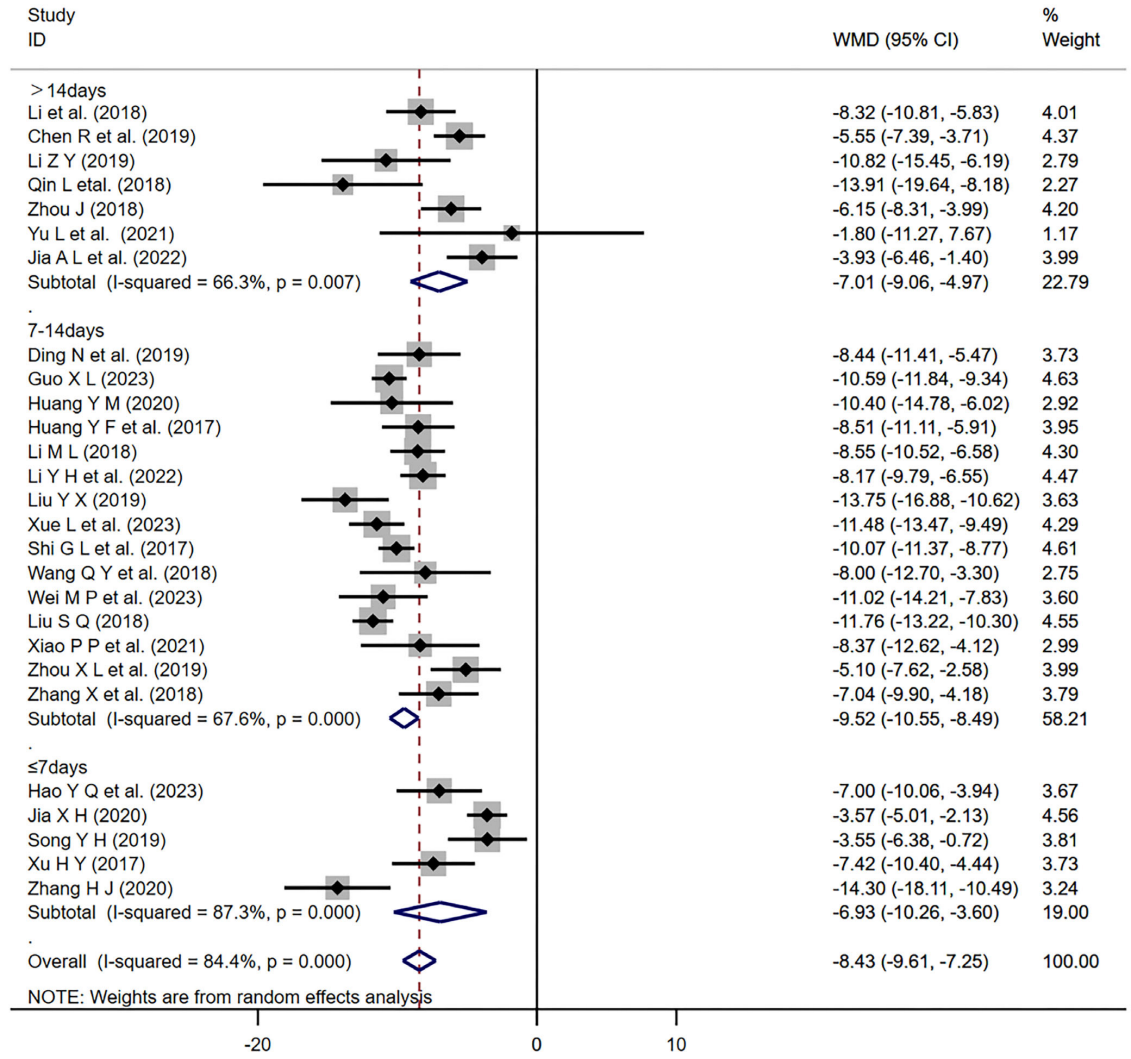
**Forest plot of treatment duration subgroup analysis of DBP.** DBP: Diastolic blood pressure; WMD: Weighted mean differences; CI: Confidence intervals.

**Figure 16.**
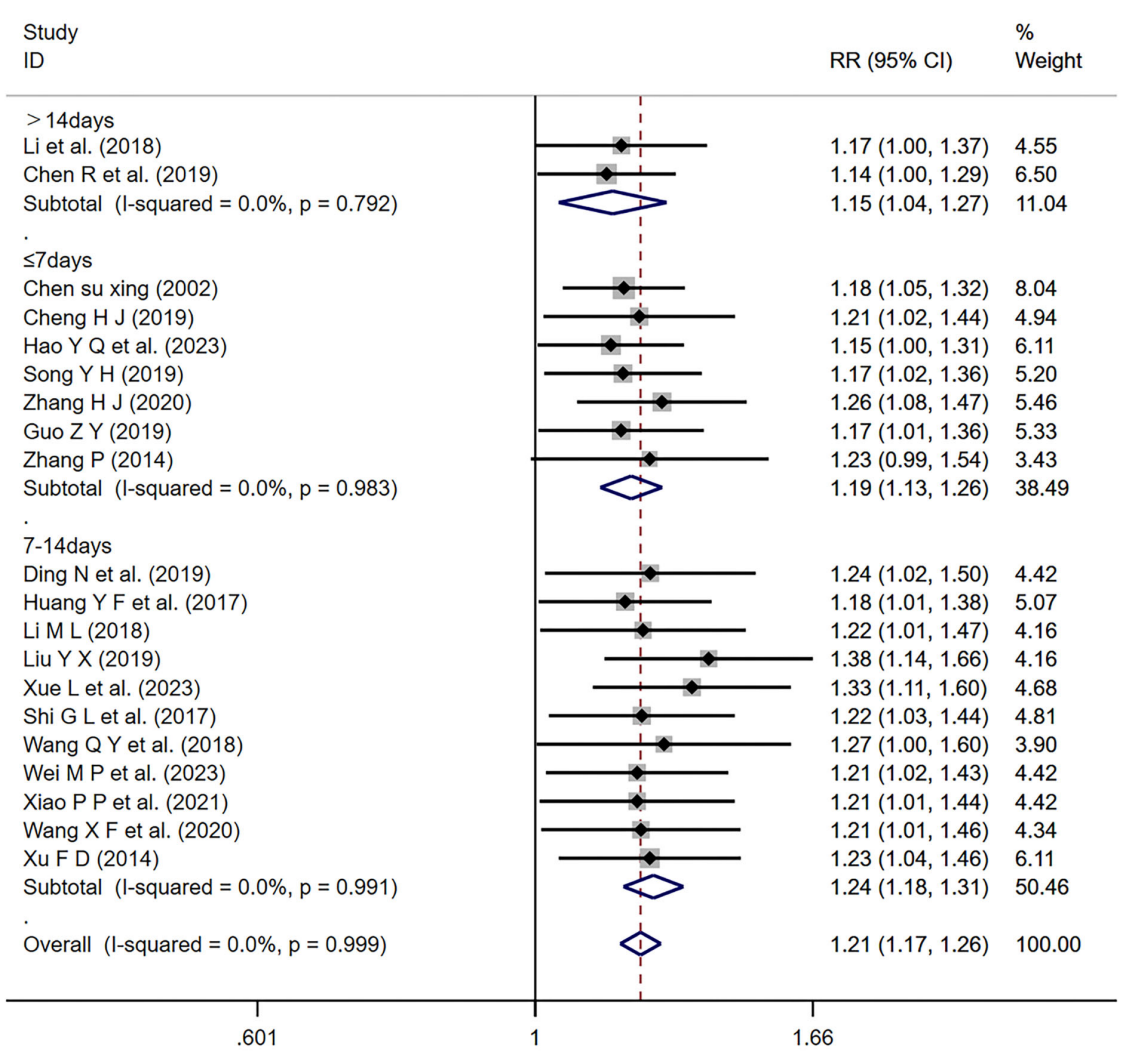
**Forest plot of treatment duration subgroup analysis of overall response rate.** RR: Relative risk; CI: Confidence intervals.

For each major outcome measure (SBP, DBP, ORR), we conducted subgroup analyses based on the duration of the treatment (up to seven days; over 14 days; 7–14 days) (Figures 14–16). The results showed that all three treatment durations had a significant effect on improving the indices as long as they were treated with the program (*P* < 0.01). Furthermore, our regression analysis indicated that changes in SBP might be influenced by different treatment durations (*P* ═ 0.042 < 0.05).

*Sensitivity analysis*

Sensitivity analyses of the key outcome indicators indicated that the findings were fundamentally reliable (Figures S1–S9).

*Publication bias*

Primary outcome measures were tested for publication bias using funnel plots, Egger’s test, or Begg’s test. The data indicated that publication biases in blood pressure changes and pregnancy outcome indicators in mothers and infants treated with combined Salvia-ligustrazine were not significant (*P* > 0.05). However, there was a notable publication bias (*P* < 0.01) for total efficiency, and our conclusions did not change after we supplemented the analysis with the eight publications using the cut-and-patch method. Additionally, there was publication bias in the maternal and infant pregnancy outcome indicators of combined Chuanxiongzine treatment (*P* < 0.05), and the conclusions remained unchanged after applying the cut-and-patch method, further validating our findings.

## Discussion

PIH represents a considerable threat to the health of both mothers and infants. In severe cases, it can precipitate placental abruption, eclampsia, cardiovascular and cerebrovascular accidents, as well as other adverse pregnancy outcomes [54]. The conventional therapeutic agents employed in such cases included magnesium sulfate and sedative antihypertensive drugs. However, the results are often unsatisfactory and accompanied by a variety of adverse effects, underscoring the urgent need for improved treatment options. Salvia-ligustrazine and ligustrazine may be suitable pharmacological options for the management of PIH. Clinical applications have shown favorable outcomes, facilitating more efficacious treatment regimens for patients diagnosed with PIH.

Based on meta-analysis, evidence suggests that the combination of Salvia-ligustrazine or Ligustrazine injections with conventional treatment is superior to the use of Labetalol, Nifedipine, and similar drugs alone in terms of reducing blood pressure, improving coagulation function, enhancing clinical efficacy, and improving maternal and infant pregnancy outcomes. Pharmacological studies have shown that Salvia miltiorrhiza acts against myocardial ischemia, atherosclerosis and thrombosis [55]. Ligustrazine is an alkaloid, and the main active substance extracted from the rhizome of Artemisia *Ligusttcum chuanxiong Hort*, family Umbelliferae, which is widely used clinically for a variety of diseases. Pharmacological reviews have established that Chuanxiongzine has antithrombotic, Anti-ischemia-reperfusion injury, and cardiovascular and cerebrovascular protective effects. Ligustrazine may exert vasodilatory effects through the activation of the adenylate cyclase (AC)/PKA cascade and inhibition of voltage-dependent L-type Ca^2+^ channels [56]. Xu et al. [57] found that Ligustrazine may improve vascular endothelial cell dysfunction by increasing in mitochondrial biosynthesis.

The results showed that treatment with Ligustrazine injections combined with antispasmodic drugs, such as magnesium sulfate or diazepam substantially reduced the number of eclamptic episodes and the severity of eclampsia. Pharmacological studies suggest that Ligustrazine protects the brain and nerves through antioxidant and anti-apoptotic pathways. Xiaoqin [58] found that Ligustrazine inhibited the production of glutamate (Glu) in the brain and promoted the production of gamma-aminobutyric acid (GABA), thus reducing neuronal excitability and inhibiting epilepsy. However, Salvia-ligustrazine did not improve eclampsia.

It was found that the injection of Salvia-ligustrazine or Ligustrazine in combination with conventional Western medicine treatment reduced side effects, such as dizziness, nausea, and liver and kidney dysfunction. Ligustrazine has been found to have biological activities, such as protecting liver and kidney function, detoxification, antipyretic effects, and immune enhancement. Cui et al. [59] found that Ligustrazine reduced cochlear ototoxicity by decreasing hearing threshold changes and reducing the expression of heat shock protein 70 and caspase-3 proteins. Ligustrazine also enhanced the phagocytosis of murine peritoneal macrophages, promoted T-lymphocyte esterase positivity, and increased serum hemolysin content, according to Daohong et al. [60].

In summary, the combination of traditional therapy with Salvia-ligustrazine or Ligustrazine injections represents a viable strategy for enhancing efficacy while minimizing adverse effects in patients diagnosed with PIH. This aligns with findings from pharmacological studies on Salvia and Chuanxiong species. Firstly, evidence indicates that the therapy is more effective in lowering blood pressure. Secondly, this therapy diminishes the likelihood of adverse pregnancy outcomes, including eclampsia, placental abruption, postpartum hemorrhage, and other complications. Furthermore, it has fewer adverse effects. The regression analysis demonstrated that both herbal injections exhibited favorable therapeutic effects after approximately one to two weeks of continuous treatment, with no significant difference between them. Prolonged treatment duration did not lead to a statistically significant enhancement of clinical outcomes. In our regression analyses, we found that SBP values appear to be affected by the duration of treatment. Specifically, our findings suggest that treatment lasting more than seven days may be more efficacious than treatment lasting up to seven days.

However, it should be noted that the study has certain limitations. While the overall heterogeneity of the articles was relatively low, the heterogeneity of ORR and pregnancy outcomes among the outcome indicators was considerable. Despite performing a thorough heterogeneity analysis, we were unable to identify the source of this heterogeneity. We speculate that the unclear randomization process in the majority of the included studies may have resulted in the omission of crucial information regarding blinding, case shedding, and other factors. This may have contributed to the observed heterogeneity in the present study. RCTs are rare, and the study was geographically limited, being almost entirely from China. This underscores the need for additional high-quality trials to provide supporting evidence for our findings.

## Conclusion

In conclusion, the available evidence supports the hypothesis that the combination of Chinese herbal injections, specifically Salvia-ligustrazine or Ligustrazine, is more effective in treating PIH than Western medicine alone. This combination is associated with a higher overall response rate and fewer adverse pregnancy outcomes. Additionally, Salvia-ligustrazine or Ligustrazine injection therapy demonstrates superior safety characteristics and offers longer-term benefits. However, further well-designed studies are needed to substantiate these findings.

## Supplemental data

Supplementary data are available at the following links: https://www.bjbms.org/ojs/index.php/bjbms/article/view/10743/3402 https://www.bjbms.org/ojs/index.php/bjbms/article/view/10743/3497

## Data Availability

All data generated during the study are presented in the article/Supplementary Material.
